# AI in Psoriatic Disease: Scoping Review

**DOI:** 10.2196/50451

**Published:** 2024-10-16

**Authors:** Richard Barlow, Anthony Bewley, Maria Angeliki Gkini

**Affiliations:** 1 Dermatology Department University Hospital Birmingham NHS Foundation Trust Birmingham United Kingdom; 2 Department of Dermatology The Royal London Hospital Barts Health NHS Trust London United Kingdom

**Keywords:** artificial intelligence, machine learning, psoriasis, psoriatic arthritis, psoriatic disease, biologics, prognostic models, mobile phone

## Abstract

**Background:**

Artificial intelligence (AI) has many applications in numerous medical fields, including dermatology. Although the majority of AI studies in dermatology focus on skin cancer, there is growing interest in the applicability of AI models in inflammatory diseases, such as psoriasis. Psoriatic disease is a chronic, inflammatory, immune-mediated systemic condition with multiple comorbidities and a significant impact on patients’ quality of life. Advanced treatments, including biologics and small molecules, have transformed the management of psoriatic disease. Nevertheless, there are still considerable unmet needs. Globally, delays in the diagnosis of the disease and its severity are common due to poor access to health care systems. Moreover, despite the abundance of treatments, we are unable to predict which is the right medication for the right patient, especially in resource-limited settings. AI could be an additional tool to address those needs. In this way, we can improve rates of diagnosis, accurately assess severity, and predict outcomes of treatment.

**Objective:**

This study aims to provide an up-to-date literature review on the use of AI in psoriatic disease, including diagnostics and clinical management as well as addressing the limitations in applicability.

**Methods:**

We searched the databases MEDLINE, PubMed, and Embase using the keywords “AI AND psoriasis OR psoriatic arthritis OR psoriatic disease,” “machine learning AND psoriasis OR psoriatic arthritis OR psoriatic disease,” and “prognostic model AND psoriasis OR psoriatic arthritis OR psoriatic disease” until June 1, 2023. Reference lists of relevant papers were also cross-examined for other papers not detected in the initial search.

**Results:**

Our literature search yielded 38 relevant papers. AI has been identified as a key component in digital health technologies. Within this field, there is the potential to apply specific techniques such as machine learning and deep learning to address several aspects of managing psoriatic disease. This includes diagnosis, particularly useful for remote teledermatology via photographs taken by patients as well as monitoring and estimating severity. Similarly, AI can be used to synthesize the vast data sets already in place through patient registries which can help identify appropriate biologic treatments for future cohorts and those individuals most likely to develop complications.

**Conclusions:**

There are multiple advantageous uses for AI and digital health technologies in psoriatic disease. With wider implementation of AI, we need to be mindful of potential limitations, such as validation and standardization or generalizability of results in specific populations, such as patients with darker skin phototypes.

## Introduction

Artificial intelligence (AI) is generally regarded as the ability of machines to simulate human intelligence, and typically refers to computers or software. Although the term AI is used daily, a standard definition is lacking. In 1950, Alan Turing [1] suggested a method to examine machine intelligence via an exercise now termed the Turing test. In this exercise, an impartial observer deemed a machine intelligent if it was indistinguishable from a human in conversation [1]. Nowadays, AI refers to the ability of a machine to communicate, reason, and operate independently in both familiar and novel scenarios in a similar manner to a human and that is not indistinguishable [2].

AI is distinct from machine learning (ML), although the 2 terms are often used interchangeably (Textbox 1) [2]. ML is a subset of AI that is related to teaching machines to automatically learn tasks from data by recognizing and inferring patterns within them [2]. Due to the growth in available patients’ medical data, ML’s potential to comprehend medical tasks has significantly increased. Algorithms can also be learned via deep learning (DL), which can be performed without labeled data sets. DL refers to a neural network with multiple layers of “neurons” that have adjustable weights (mathematical functions) [2], with ML to train or test data across its network for improved accuracy and performance.

**Essential terminology in the field of machine learning and artificial intelligence.**

- Artificial intelligence: The ability of machines, such as computers, to simulate human intelligence.
- Machine learning: Algorithms and statistical models that are programmed to learn from data, therefore recognizing and inferring patterns within them. This enables computers to perform specific tasks without explicit instructions from a human operator.
- Deep learning: Refers to a neural network with multiple layers of “neurons” that have adjustable weights (mathematical functions), with machine learning to train or test data across its network for improved accuracy and performance.

Both the US Department of Health and Human Services [3] and the European Union [4] have outlined potential future roles and implementation of AI within health care. The National Health Service in the United Kingdom has also identified AI as a current and future priority; the 2019 Topol review [5] published by Health Education England includes AI as one of 3 key digital health technologies. The report also details how to prepare the health care workforce to deliver a digital future as a response to keeping up with the increasing demands of our expanding population (Textbox 2) [5].

**Digital health technologies.**

- Genomics (reading and writing the genome)
- Digital medicine (telemedicine, apps, sensors and wearables, and virtual reality)
- Artificial intelligence (speech recognition, natural language processing, and robotics)

AI has broad applications within medical settings (Textbox 3), all of which need careful consideration regarding appropriate clinical governance [6]. Many of these applications naturally lend themselves to dermatology given the visual nature of the specialty and the large data sets already established (Textbox 4) [7,8]. Further steps forward have been taken in screening and diagnosing of melanoma and nonmelanoma skin cancers. Numerous apps and digital platforms, using dermoscopic or clinical images, have been already available although their sensitivities and specificities vary. More recently, there is growing interest in the use of AI in inflammatory skin diseases [9].

**Artificial intelligence (AI) in health care.**

- AI-assisted robotic surgery
- Virtual nursing assistants
- AI-assisted medical diagnoses
- Medical image analysis
- Drug discovery
- Automated workflow assistance
- Fraud detection
- Medical data security
- Medical risk prediction
- Clinical trials
- Personalized treatment
- Improve gene editing

**Uses of artificial intelligence in dermatology.**

- Skin cancer diagnosis
- Onychomycosis assessment
- Ulcer assessment
- Predicting skin sensitization substances
- Novel applications in pathology and gene expression profiling
- Psoriasis disease and other inflammatory skin diseases

Psoriatic disease is a common, chronic, systemic inflammatory condition with a significant impact on a patient’s quality of life. Common comorbidities include psoriatic arthritis, cardiovascular disease, metabolic syndrome, and psychiatric or psychosocial impact. More recently researched comorbidities such as liver fibrosis and renal disease also exist [10]. Concerning is the fact that these comorbidities can be found also in the US pediatric population [11]. Advanced systemic treatments, including biologics and small molecules can improve comorbidities [12].

Globally, the problem of delayed and or incorrect diagnosis of psoriasis remains. Long waiting lists and pressure to prioritize potential malignant lesions also prolong patients accessing appropriate specialist dermatology care. In areas of limited resources, access to dermatology can also be a challenge. Identifying the most appropriate treatment for each individual remains an unmet goal, although recently there have been updates in the field of genomics and personalized medicine [13,14]. Therefore, AI offers progress in both directions, diagnostics and management (Textbox 5).

**Roles for artificial intelligence in psoriasis evaluation using skin images.**

**Diagnosis**

- Identification and differential diagnosis of psoriasis lesions
- Assessment of severity

**Clinical management**

- Prediction of complications
- Treatment
- Discovery of new biomarkers and drug targets

Our objective was to provide an up-to-date literature review on the use of AI in psoriatic disease, including diagnostics and clinical management as well as addressing the limitations in applicability.

## Methods

We conducted a literature search from the databases MEDLINE, PubMed, and Embase using the keywords “AI AND psoriasis OR psoriatic arthritis OR psoriatic disease,” “ML AND psoriasis OR psoriatic arthritis OR psoriatic disease,” and “prognostic model AND psoriasis OR psoriatic arthritis OR psoriatic disease” until June 1, 2023. Reference lists of relevant papers were also cross-examined for other papers not detected in the initial search. RB and MAG screened papers and discrepancies were reviewed by AB. For articles that met our inclusion criteria, the study characteristics and outcomes were abstracted using a spreadsheet developed by the research team. The data abstraction was conducted by RB. The spreadsheet was reviewed and discussed by RB, MAG and AB with key findings categorized under ‘Diagnosis and assessment of severity’ and ‘Clinical management’.

## Results

**Figure 1:**
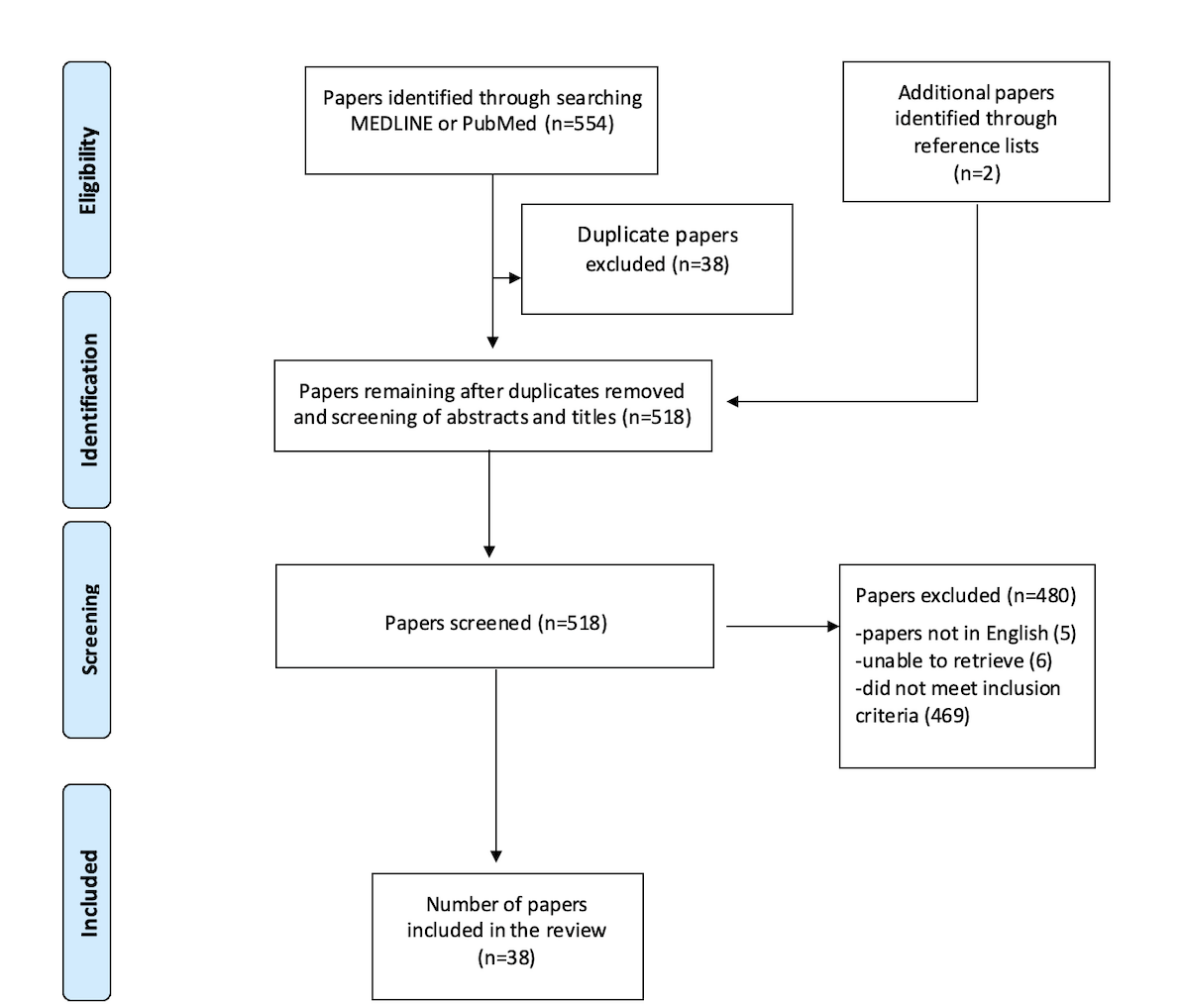
PRISMA (Preferred Reporting Items for Systematic reviews and Meta-Analyses) flowchart of methodology to identify included studies.

Figure 1 demonstrates the flowchart of methodology. The search strategy and screening yielded a total of 38 relevant papers. These included a range of observational, interventional and descriptive studies across a multitude of populations and datasets.

### Diagnosis and Assessment of Severity

Correct, timely diagnosis is the first step of management in psoriasis; distinguishing it from other similar disorders can be particularly challenging for clinicians and AI alike.

Google has recently launched its own AI-tool for differentiating between 288 conditions and is undergoing validation. Users can upload 3 well-lit images of the skin, hair, or nail of concern from different angles. The tool then asks questions related to personal history, and combining this with the image, it suggests a list of possible causative conditions. The user is then signposted to similar matching images and dermatologist-reviewed information [15]. This system is underpinned by a DL system formed from a data set containing over 16,000 pictures of skin disorders. The system was shown to be noninferior to 6 board-certified US dermatologists and superior to 6 general practitioners [16].

On the scalp, psoriasis and seborrheic dermatitis can look very similar. Multispectral imaging allows pictographic data to be collected from regions of the electromagnetic spectrum not visible to the human eye. Historically speaking, processing such vast data can be time-consuming, with the equipment being bulky and cumbersome. A smartphone-based multispectral imaging system in conjunction with ML has been used in South Korea to differentiate between psoriasis and seborrheic dermatitis of the scalp—allowing for swifter data interpretation and AI-based diagnosis. The authors used a small handheld multispectral camera, which displayed results on a smartphone screen for diagnosis and monitoring. Kim et al [17] achieved a sensitivity of 65% to 75% and specificity of 70% to 80% in psoriasis diagnosis of the scalp versus seborrheic dermatitis. Moreover, the ML methods yielded better outcomes versus conventional methods.

Psoriasis can also be differentiated from other similar looking inflammatory disorders. Zhao et al [18] classified 8021 images of 9 skin conditions using convolutional neural networks in psoriasis versus nonpsoriasis. Images included were lichen planus, lupus erythematosus, basal cell carcinoma, squamous cell carcinoma, atopic dermatitis, pemphigus, seborrheic keratosis, and psoriasis from a cohort of patients from a Chinese hospital. Their algorithm was superior to 25 Chinese dermatologists when tested on 100 new images. They reported a misdiagnosis rate of 3% compared to 27% by dermatologists.

Further to diagnosis, severity assessment is key to disease monitoring as well as determining which treatments are indicated. Shrivastava et al [19] used ML to analyze 540 images of skin (270 affected and 270 nonaffected images) in a cohort of 30 patients of Indian heritage with psoriasis. Principal component analysis was used to condense the data without losing important data points. This, in combination with computer-aided diagnosis, was used to define 3 main components of each image: higher order spectra features, texture features, and color features. This type of ML, when combining the 3 features, was proven to be highly effective with a classification accuracy of approximately 99% [19].

More sophisticated assessments such as psoriasis area and severity index (PASI) is widely used and present an additional challenge for AI with textural changes and thickness featuring in the scoring. This is further complicated by the regional scores, which require AI to determine which part of an image is unaffected as a percentage of overall body surface area. Huang et al [20] used a database of 14,096 images from a cohort of 2367 Chinese patients with psoriasis to estimate PASI. Multiview enhancement block (images from different angles) was implemented to allow all facets of the PASI score to be estimated. These images were then processed with convolutional neural network to extract specific features. Despite the large subjectivity of PASI scoring in clinical practice, the DL method used by the authors was comparable to PASI scores calculated by 43 dermatologists and has been successfully used in 18 different sites via the use of an app [20]. This has important ramifications for how we may deal with patients in more remote settings and those patients with less flexibility, where severity needs to be objectively scored.

Okamoto et al [21] developed this concept further via a technique termed “single-shot” PASI. The authors used 705 images of psoriasis, expanded with data augmentation techniques. Expert scoring was used as teacher data for the system to learn from. This DL system was then able to accurately assess psoriasis severity from a single photograph only, reducing interuser variability and increasing efficiency [21].

### Clinical Management

Despite an abundance of classic and novel treatments for psoriasis, including biologics and small molecules, there are vast unmet needs in the management of psoriatic disease. A significant number of patients may not respond to a specific treatment primarily or secondarily or may develop adverse events. Therefore, we are still unable to predict the right treatment for the right patient. Available guidelines can help us select an appropriate biologic agent [22], although understanding and predicting outcomes is much more challenging. Biomarkers that could predict response to biologic therapy would be ideal for clinical care, although a lack of robust experimental data means no consensus exists yet toward application in clinical practice [23]. The use of AI to combine genotypic and phenotypic characteristics of patients with psoriasis to identify the most appropriate treatment is starting to emerge.

Emam et al [24], a Danish group in 2020, analyzed data from 681 patients with psoriasis from a national registry. A total of 6 different ML techniques were used to identify patterns from demographic and clinical data: generalized linear model, support vector machine, decision tree, random forest, gradient-boosted trees, and DL. Treatment outcomes were able to be predicted with high accuracy and less than 18% classification error, with data that are routinely available to clinicians. The generalized linear model was found to be the most accurate. Additionally, the model was able to identify characteristics associated with prolonged successful treatment, including but not limited to the age of 23 years and older at the time of diagnosis, baseline dermatology life quality index ≥16, baseline PASI ≥94, and weight ≤98.9 kg [24]. The study was limited by its retrospective nature, and it did not take into account access to medication, which could have limited duration of treatment.

Nielsen et al [25] used the same registry to retrospectively predict the most suitable biological therapy for patients with psoriasis. The authors found that gradient-boosted decision trees, a specific type of ML, performed significantly better than logistic regression for the prediction of specific biologic therapy. This technique was able to predict discontinuation of a given biologic within the first year of treatment with an accuracy of 62.9% to 67.6% [25].

AI is a natural fit to interpret the considerable amount of data that naturally accumulates as health care becomes more digitalized, which can often be dynamic through real-time capture. “Big data” is therefore well suited to interpretation via ML to draw patterns that may deepen our understanding of treatment trajectories and pathophysiology, taking health care one step closer to personalized medicine.

Bragazzi et al [26] performed a systemic review to map the current use of ML for big data analysis in psoriatic disease; 26 papers met the inclusion criteria. ML algorithms were shown to accurately extract patterns on predicting psoriatic arthritis from epidemiological registries, molecular databases, and different smartphone health applications. The authors did however highlight that we should be mindful of data protection, confidentiality, and association biases [26].

Furthering genetic understanding of psoriasis paves the way for future treatment options as well as aiding in diagnosis and prediction of progression. Genetic medicine provides an enormous amount of data which can also benefit from ML interpretation. Using genetic markers to select those who are at the most risk of developing psoriatic arthritis could have a large potential for reducing long-term morbidity. With each new gene identified comes possible new targets for therapy—more of these are being identified through increasingly sophisticated data analysis [27]. Encouragingly, AI has also been shown to predict psoriasis highly accurately from microarray-based gene expression profiles [27].

Genome-wide association studies provide enormous amounts of complex data that can be swiftly interpreted via ML techniques. Patrick et al [28] used ML (including random forest, conditional inference forest, shrinkage discriminant analysis, and elastic net regression) to identify 9 novel loci for psoriasis following evaluation of more than 7000 genotyped patients with psoriasis provided by genome-wide association studies. Love et al [29] analyzed data on 2318 patients with psoriatic arthritis to identify 31 psoriatic arthritis-related predictors. A single psoriatic arthritis code had a positive predictive value of 57% (95% CI 0.55-0.58], which increased to 90%-93% following natural language processing.

We should be mindful that AI is susceptible to various bias, and ethical aspects need to be considered with the patient as reference. Textbox 6 [30,31] outlines broader disadvantages of AI applicable to psoriasis, and by contrast some of the advantages as well.

**Advantages and disadvantages of artificial intelligence (AI).**

**Advantages**

- Use in repetitive and time-consuming tasks
- Use in tasks with poor interobserver reliability
- Creative diagnostics via AI
- Applications for resource-limited settings
- Interpretation of big data

**Disadvantages**

- Choice of predictive model
- Generalizability
- Standardization
- Interpretability
- Data requirements
- Acceptance
- Liability

## Discussion

### Overview

In the future, AI will certainly take health care closer to personalized medicine. As research interests continue to grow, we will see an increase in application of AI, and this will translate to greater benefits to our patients. As described in Textbox 6, AI can be used to reach greater numbers of patients—particularly those in remote and resource-limited settings.

Gaps do remain in the literature and there is a need for robust clinical governance when handling large volumes of patient data, which limits AI’s applicability to current clinical practice. Fortunately, the International Psoriasis Council has agreed upon 36 statements around psoriasis and telemedicine relating to diagnosis and treatment, which will continue to progress the frontier [32].

Despite the advances in the field of AI and psoriatic disease, AI is still not a panacea. Further validated models are needed to assess its role in both the diagnosis and the management of psoriatic disease. Dermatologists can play a crucial role in the evolution of AI and relevant training is required as well as cooperation with other specialties, such as data scientists. In the future, AI and ML may be able to predict the clinical course of the disease and, in combination with molecular studies, may even be able to guide our choice of treatment although acceptance by patients will need to be considered.

Moreover, teledermatology has gained significant popularity after the COVID-19 pandemic. Combining teledermatology with AI in psoriatic disease could indeed transform our current practice, as suggested by the International Psoriasis Council in their statements concerning remote monitoring of patients [32].

### Limitations

One of the challenges of AI in psoriatic disease resides with interpreting 3D aspects missed in imaging, such as thickness of plaques for calculating the PASI. While multiview enhancement block may combat this to some extent, most algorithms only assess area, erythema, and/or scale [33], making an accurate PASI assessment difficult to achieve.

Additionally, AI diagnostics seem to be less accurate on dark skin color than lighter skin, even when trained on equal numbers of images [34,35]. More work is needed to optimize accuracy when using AI as a diagnostic tool in individuals with darker skin phototypes. Of note, most data sets are based on European and Australian teaching sets, which limits the ability of ML to perform on individuals with darker skin phototypes [36].

An Australian survey of over 4000 individuals demonstrated that people experiencing accuracy was consistently the most important factor in using AI and reducing costs the least important. Notably, 3558 (80%) responders valued continued human contact more than other factors [37]. Another international survey of dermatologists found that only 292 (23%) had good knowledge on the subject while 116 (17%) of 680 hospital-based dermatologist were fearful of the technology, although most agreed that dermatology would provide benefit overall [38].

Further, issues of choice regarding the right predictive model exist. These include small sample size of studies and variation in systems used, which make comparisons of studies challenging.

There are multiple guidelines and recommendations in the literature, which give suggestions on how to use AI safely, including methodologies for reporting on studies that use AI [39]. Interestingly, there is little mention of any such adherence in many AI and ML studies, although this likely represents unawareness rather than poor methodology.

AI is coming to the forefront of governing bodies. In June 2023, the European Parliament begun negotiations on the AI Act; a framework for incorporating AI safely into health care [40].

### Conclusions

Future management of psoriatic disease will use diagnostic and therapeutic tools that are tailored to a group of patients with common characteristics, taking health care closer to precision medicine. As for dermatologists, it is crucial to embrace these new technologies and familiarize with them, in order to provide the best possible care to our patients, although we should be mindful that there remains a demand for human-human, face-to-face interaction.

## Appendix

Multimedia Appendix 1 PRISMA (Preferred Reporting Items for Systematic reviews and Meta-Analyses) checklist.

## Abbreviations

AI: artificial intelligence
DL: deep learning
ML: machine learning
PASI: psoriasis area and severity index
